# Co-Circulating Monkeypox and Swinepox Viruses, Democratic Republic of the Congo, 2022

**DOI:** 10.3201/eid3004.231413

**Published:** 2024-04

**Authors:** Thierry Kalonji, Emile Malembi, Jean Paul Matela, Toutou Likafi, Eddy Kinganda-Lusamaki, Emmanuel Hasivirwe Vakaniaki, Nicole A. Hoff, Amuri Aziza, Francisca Muyembe, Joelle Kabamba, Tine Cooreman, Béatrice Nguete, Danae Witte, Ahidjo Ayouba, Nicolas Fernandez-Nuñez, Stijn Roge, Martine Peeters, Sydney Merritt, Steve Ahuka-Mundeke, Eric Delaporte, Elisabeth Pukuta, Joachim Mariën, Eugene Bangwen, Steven Lakin, Charles Lewis, Jeffrey B. Doty, Laurens Liesenborghs, Lisa E. Hensley, Andrea McCollum, Anne W. Rimoin, Jean Jacques Muyembe-Tamfum, Robert Shongo, Didine Kaba, Placide Mbala-Kingebeni

**Affiliations:** Program National Lutte Contre MPX-VHF, Kinshasa, Democratic Republic of the Congo (T. Kalonji, E. Malembi, R. Shongo);; Province Health Division, Tshuapa, Democratic Republic of the Congo (J.P. Matela, T. Likafi);; Institute National de Recherche Biomedical, Kinshasa (E. Kinganda-Lusamaki, E.H. Vakaniaki, A. Aziza, F. Muyembe, S. Ahuka-Mundeke, E. Pukuta, J.J. Muyembe-Tamfum, P. Mbala-Kingebeni);; Cliniques Universitaires de Kinshasa, Kinshasa (E. Kinganda-Lusamaki, F. Muyembe, S. Ahuka-Mundeke, J.J. Muyembe-Tamfum, P. Mbala-Kingebeni);; University of Montpellier, IRD, INSERM, Montpellier, France (E. Kinganda-Lusamaki, A. Ayouba, N. Fernandez-Nuñez, M. Peeters, E. Delaporte);; University of California, Los Angeles, California, USA (N.A. Hoff, S. Merritt, A.W. Rimoin);; Centers for Disease Control and Prevention, Atlanta, Georgia, USA (J. Kabamba, J.B. Doty, A. McCollum);; University of Antwerp, Antwerp, Belgium (T. Cooreman, J. Mariën);; Kinshasa School of Public Health, Kinshasa (B. Nguete, D. Kaba);; University of Florida, Gainesville, Florida, USA (D. Witte);; Institute of Tropical Medicine, Antwerp (S. Roge, E. Bangwen, L. Liesenborghs);; US Department of Agriculture, Manhattan, Kansas, USA (S. Lakin, C. Lewis, L.E. Hensley)

**Keywords:** monkeypox virus, mpox, swinepox virus, swinepox, orthopoxvirus, infectious diseases, outbreak investigation, One Health, sexually transmitted infections, vector-borne infections, viruses, zoonoses, Democratic Republic of the Congo

## Abstract

In September 2022, deaths of pigs manifesting pox-like lesions caused by swinepox virus were reported in Tshuapa Province, Democratic Republic of the Congo. Two human mpox cases were found concurrently in the surrounding community. Specific diagnostics and robust sequencing are needed to characterize multiple poxviruses and prevent potential poxvirus transmission.

Until recently, the Democratic Republic of the Congo (DRC), located in an mpox-endemic region, has had the highest global burden of mpox (*1*). Mpox is caused by the monkeypox virus (MPXV), which belongs to the *Orthopoxvirus* genus of the Poxviridae family. Within Poxviridae, 10 other genera are known to infect animals and reptiles (*2*), including *Suipoxvirus* (*3*). The only known species of *Suipoxvirus* causes swinepox, a pox-like disease in pigs.

Two genetically distinct clades of MPXV cause disease in humans. Clade I (formerly Congo Basin clade) is endemic in Central Africa, and outbreaks are thought to result mainly from zoonotic spillover; squirrels or other small mammals are suspected reservoirs or hosts (*4*; J. Mariën et al., unpub. data, https://doi.org/10.21203/rs.3.rs-414280/v2). Clade II (formerly West African clade) is endemic in West Africa; suspected reservoirs and transmission routes are historically similar to those of clade I. In 2022, MPXV subclade IIb spread beyond West Africa, causing an unprecedented global outbreak, leading the World Health Organization to declare mpox as a Public Health Emergency of International Concern (*5*).

During 2011–2015, the incidence of MPXV cases in DRC’s Tshuapa Province increased compared with the incidence during 1981–1986 in similar geographic areas (*6*). This observation is not unique, suggesting expanding spillover rates of MPXV clade I into the human population and highlighting the possibility of increasing human-to-human virus transmission (*1*,*4*).

Confirmation of suspected MPXV cases in DRC is hindered by logistical difficulties, such as complications involving sample collection and lack of available diagnostic laboratories in remote regions (*4*). Reported increases in mpox cases without well-defined origins suggest that a One Health investigation might be a more informative strategy. As part of this approach, investigations by DRC’s National Program for Mpox and Viral Hemorrhagic Fevers (PNLMPX-VHF) and its partners, including the US Centers for Disease Control and Prevention (CDC) and Belgium Institute of Tropical Medicine, have begun sampling mammals in areas with mpox cases and animals manifesting pox-like lesions (*7*). Previous sampling focused on wildlife and small animals but has recently been expanded to include domestic animals because animal husbandry is widespread throughout DRC (*8*). Yet, areas with livestock have not been regularly surveilled for health, hygiene, or suspected viruses, including MPXV. Close proximity of humans and animals provides an environment conducive to virus spillover (*9*). We report on the co-circulation of MPXV and swinepox virus in the same locality within DRC.

## The Study

In early September 2022, provincial authorities in Tshuapa, a heavily forested province in northwest DRC, alerted PNLMPX-VHF regarding the deaths of pigs in the Boende health zone that manifested vesicular, pox-like lesions. Authorities also reported several suspected mpox cases among farm owners and residents in the area; clinical samples were not collected from those persons. Tshuapa is considered an MPXV-endemic area and has a robust surveillance program through PNLMPX-VHF, Kinshasa School of Public Health, and CDC. Through this program, suspected mpox case samples are sent to DRC’s reference laboratory, the Institute National de Recherche Biomedical (INRB), for testing and, when requested, sequencing (*6*).

**Figure 1:**
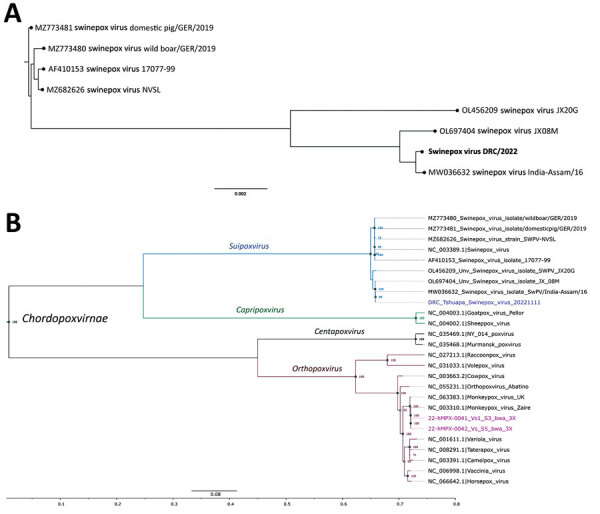
Phylogenetic analysis of swinepox virus (A) and monkeypox virus (B) in study of co-circulating viruses, Democratic Republic of the Congo, 2022. Poxvirus sequences for comparison were obtained from GenBank. Trees were constructed by using the maximum-likelihood method and the general time reversal substitution model with gamma distribution and proportion of invariable sites. In panel A, bold text indicates the swinepox sequence from this study; in panel B, magenta-colored text indicates monkeypox virus sequences and blue text indicates swinepox virus sequence from this study. The monkeypox virus samples belong to clade I and correspond to those previously described (*11*). Scale bars indicate nucleotide substitutions per site. DRC, Democratic Republic of the Congo.

After the alert, during September 9–19, two pigs with pox-like lesions were identified at a domestic farm where pigs roamed freely. The first diseased pig had died on September 1; no samples had been collected. On September 10, the second pig, which had predominant umbilicated nodular dermatitis between the ears and on the face and chest, died. We collected a vesicle swab sample from the cadaver for analysis at INRB. MPXV PCR (*10*) results were negative, and we sequenced the sample by using an Illumina platform (Illumina, https://www.illumina.com) probe enrichment-based protocol and a custom-made virus research panel (Twist Biosciences, https://www.twistbioscience.com). We used the open source bioinformatics pipeline, GeVarLi (https://forge.ird.fr/transvihmi/nfernandez/GeVarLi), to clean and align sequences and assign virus lineages. We completed de novo sequence assembly by using SPAdes Genome Assembler (https://github.com/ablab/spades). Sequencing results and phylogenetic analysis indicated that the second pig had been infected with swinepox virus (Figure 1, panel A) (*11*).

After identifying a swinepox-positive pig, we launched an expanded poxvirus investigation on October 2. In the Boende health zone, we identified 16 suspected human mpox cases, all occurring within 400 m of the farm that had a confirmed swinepox case. None of the persons with suspected mpox reported having mpox vaccinations, nor did they have scars indicating past mpox or variola vaccination. We collected skin swab, oropharyngeal lesion swab, and venous blood samples from case-patients with suspected active (n = 2) and convalescent (n = 4) MPXV infections and analyzed those samples at INRB. The 2 suspected active case-patients (both children) were MPXV positive. Sequencing confirmed infection with MPXV; however, we did not detect swinepox co-infection (Figure 1, panel B). We deposited the 2 MPXV sequences in the GISAID database (https://www.gisaid.org; accession nos. EPI_ISL_18857033–4). We determined that the farm owner, not realizing his pigs were sick, permitted his 2 children to directly contact the pigs during feeding without protection. Both children exhibited mild symptoms of fever, colds, headaches, and physical asthenia and developed a rash and papules 12 days after disease onset in the first pig. Both MPXV PCR-positive children recovered without complications; in addition, the family reported that they had previously recovered from PCR-confirmed MPXV. No epidemiologic link was established between the mpox-positive children and the swinepox-positive pig.

**Figure 2:**
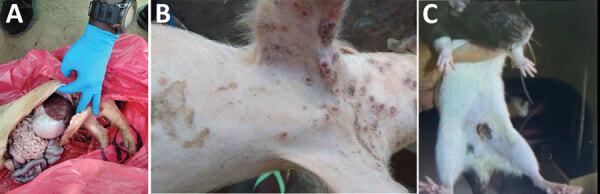
Animals with mpox-like lesions in study of co-circulating monkeypox and swinepox viruses, Democratic Republic of the Congo, 2022. A, B) Mpox-type lesions observed on internal tissues (A) and on the skin (B) of a pig. C) Lesion on the skin of a rodent that was found in the same geographic area as the pig.

We returned to the area to identify additional pigs with symptoms of pox-like virus infections and found 4 suspected cases, of which 3 were in a neighboring farm. All pigs with suspected infections had pox-like skin lesions on the face, mouth, or tongue and had liver pathology similar to clinical manifestations of pox-like virus infections. A third pig from the original farm died on October 24; we collected lesion crust, vesicle, and rectal swab samples and internal organs where vesicular lesions were noted (Figure 2, panels A, B). Small mammals found in the vicinity of the original farm and that farmer’s house also showed similar skin lesions (Figure 2, panel C). We collected lung, spleen, kidney, liver, oral, and rectal swab samples from 28 small mammals (shrews and black rats [*Rattus rattus*]) for analysis at INRB; 2 rats exhibited lesions. All samples tested negative for MPXV by PCR, and no additional sequencing was performed.

Concurrently, medical staff reported periodic human outbreaks of suspected MPXV infections and wild and domesticated animal deaths in other health zones. In addition, outbreaks of pox-like disease in pigs preceded confirmed human mpox cases in the Bolomba health zone of Equateur Province in 2008 and the Masi-Manimba health zone of Kwilu Province in 2022 (*12*). Although no samples were collected from sick animals during those outbreaks, the overall frequency of reports warrants future use of One Health approaches in all investigations of pox-like diseases, regardless of the initial species in which the lesions are observed.

## Conclusions

In environments endemic for zoonotic pathogens such as MPXV, the role of animal reservoirs and hosts must be considered in surveillance and outbreak control. Reports of multiple poxviruses (vaccinia virus, pseudocowpox virus, MPXV) co-circulating within a community are rare and have been primarily confined to Brazil (*2*,*13*). In addition to mpox, and now swinepox, DRC has reported cases of other poxviruses, including molluscum contagiosum and tanapox (*14*). The appearance of multiple poxviruses in the same region indicates sensitive, specific diagnostics and robust sequencing capacity are needed to characterize those agents, better understand poxvirus epidemiology, and prevent potential poxvirus transmission.

## References

[1] Bunge EM, Hoet B, Chen L, Lienert F, Weidenthaler H, Baer LR, et al. The changing epidemiology of human monkeypox-A potential threat? A systematic review. PLoS Negl Trop Dis. 2022;16:e0010141. 10.1371/journal.pntd.0010141 35148313 PMC8870502

[2] Oliveira GP, Rodrigues RAL, Lima MT, Drumond BP, Abrahão JS. Poxvirus host range genes and virus–host spectrum: a critical review. Viruses. 2017;9:331. 10.3390/v9110331 29112165 PMC5707538

[3] Afonso CL, Tulman ER, Lu Z, Zsak L, Osorio FA, Balinsky C, et al. The genome of swinepox virus. J Virol. 2002;76:783–90. 10.1128/JVI.76.2.783-790.2002 11752168 PMC136851

[4] Rimoin AW, Mulembakani PM, Johnston SC, Lloyd Smith JO, Kisalu NK, Kinkela TL, et al. Major increase in human monkeypox incidence 30 years after smallpox vaccination campaigns cease in the Democratic Republic of Congo. Proc Natl Acad Sci U S A. 2010;107:16262–7. 10.1073/pnas.1005769107 20805472 PMC2941342

[5] World Health Organization. WHO Director-General’s statement at the press conference following IHR Emergency Committee regarding the multicountry outbreak of monkeypox—23 July 2022 [cited 2023 Jun 20]. https://www.who.int/director-general/speeches/detail/who-director-general-s-statement-on-the-press-conference-following-IHR-emergency-committee-regarding-the-multi--country-outbreak-of-monkeypox--23-july-2022

[6] Whitehouse ER, Bonwitt J, Hughes CM, Lushima RS, Likafi T, Nguete B, et al. Clinical and epidemiological findings from enhanced monkeypox surveillance in Tshuapa Province, Democratic Republic of the Congo during 2011–2015. J Infect Dis. 2021;223:1870–8. 10.1093/infdis/jiab133 33728469

[7] Centers for Disease Control and Prevention. Mpox in animals and pets. January 4, 2023 [cited 2023 March 13]. https://www.cdc.gov/poxvirus/mpox/veterinarian/mpox-in-animals.html

[8] Bosworth A, Wakerley D, Houlihan CF, Atabani SF. Monkeypox: An old foe, with new challenges. Infect Prev Pract. 2022;4:100229. 10.1016/j.infpip.2022.100229 35847384 PMC9283547

[9] Vora NM, Hannah L, Walzer C, Vale MM, Lieberman S, Emerson A, et al. Interventions to reduce risk for pathogen spillover and early disease spread to prevent outbreaks, epidemics, and pandemics. Emerg Infect Dis. 2023;29:1–9. 10.3201/eid2903.221079 36823026 PMC9973692

[10] Li Y, Zhao H, Wilkins K, Hughes C, Damon IK. Real-time PCR assays for the specific detection of monkeypox virus West African and Congo Basin strain DNA. J Virol Methods. 2010;169:223–7. 10.1016/j.jviromet.2010.07.012 20643162 PMC9628942

[11] Berthet N, Descorps-Declère S, Besombes C, Curaudeau M, Nkili Meyong AA, Selekon B, et al. Genomic history of human monkey pox infections in the Central African Republic between 2001 and 2018. Sci Rep. 2021;11:13085. 10.1038/s41598-021-92315-8 34158533 PMC8219716

[12] World Health Organization. RD Congo: rapport de mission d’investigation et riposte à l’épidémie de monkey pox à Djoa, zone de santé de Bolomba. 2008 [cited 2023 Jun 23]. https://reliefweb.int/report/democratic-republic-congo/rd-congo-rapport-de-mission-dinvestigation-et-riposte-%C3%A0-l%C3%A9pid%C3%A9mie

[13] Luques MN, Oliveira RL, Hir S, Nunes DDS, Higa LM, Mendonça AF, et al. Co-circulation of vaccinia and monkeypox viruses in rural areas of Brazil: Importance of differential molecular diagnosis. Travel Med Infect Dis. 2023;53:102578. 10.1016/j.tmaid.2023.102578 37088362 PMC10122557

[14] Monroe BP, Nakazawa YJ, Reynolds MG, Carroll DS. Estimating the geographic distribution of human Tanapox and potential reservoirs using ecological niche modeling. Int J Health Geogr. 2014;13:34. 10.1186/1476-072X-13-34 25255815 PMC4189193

